# Supplementary material for: Can non-destructive DNA extraction of bulk invertebrate samples be used for metabarcoding?
Source: PeerJ. 2018 Jun 13;6:e4980. doi: 10.7717/peerj.4980 (PMC6004113; doi:10.7717/peerj.4980)
Supplement: Table S1 — Species are presented as least (left) to most (right) scelrotized. The success of non-destructive extraction for standard DNA barcoding PCR is indicated by the ‘ +’ symbol (‘ −’ for no PCR product), while the success of the second Chelex extraction on the same material is indicated in parenthesis. [file peerj-06-4980-s001.docx]

|  |  | **Gastropoda** | **Diptera** | **Odonata** | **Coleoptera** |
| --- | --- | --- | --- | --- | --- |
|  |  | ***Physa acuta* (Physidae)** | ***Procladius villosimanus***  **(Chironomidae)** | ***Ischnura heterosticta***  **(Coenagrionidae)** | ***Necterosoma* sp.**  **(Dytiscidae)** |
| **Incubation time** | **Preparation** | **Whole animal** | **Whole animal (Larvae)** | **Leg (Nymph)** | **Whole animal**  **(Adult)** |
| 1hr | Ethanol removal | + (+) | + (+) | + (+) | + (+) |
|  | Overnight in TE Buffer | + (-) | + (+) | + (+) | + (+) |
| 3hr | Ethanol  removal | + (-) | + (+) | + (+) | + (+*) |
|  | Overnight in TE Buffer | + (-) | + (+) | + (+) | + (+*) |

* extraction using crushed legs failed, but 2hr incubation of whole animal (with proteinase K) was successful.
