# Supplementary material for: Can non-destructive DNA extraction of bulk invertebrate samples be used for metabarcoding?
Source: PeerJ. 2018 Jun 13;6:e4980. doi: 10.7717/peerj.4980 (PMC6004113; doi:10.7717/peerj.4980)
Supplement: Table S2 — The success of extractions is indicated by the ‘ +’ symbol (‘ −’ for no PCR product). [file peerj-06-4980-s002.docx]

| Order (sceloritization details) | Family/Order | Species | Material for extraction | |  | 30 min incubation in extraction buffer (GenBank Accession) | |  | 1 hr incubation in extraction buffer (GenBank Accession) | |
| --- | --- | --- | --- | --- | --- | --- | --- | --- | --- | --- |
|  |  |  | Extraction 1 | Extraction 2 |  | Extraction 1 | Extraction 2 |  | Extraction 1 | Extraction 2 |
| Acarina | Hydrachnidae | *Ctya* sp. | whole animal | whole animal |  | |  | + (MG976102) | | - |
| (moderate) | Parasitidae | *Pergamasus quisquiliarum* | whole animal | whole animal |  | |  | + (MG976203) | | - |
|  | Hydrachnidae | Hydrachnidae sp. | whole animal | whole animal |  | |  | - | | - |
|  | Trombidiformes | Trombidiformes sp. | whole animal | whole animal |  | |  | - | | - |
|  | Hydryhantidae | Hydryhantidae sp. | whole animal | whole animal |  | |  | + (MG976100) | | + |
|  | Bdellidae | Bdellidae sp. | whole animal | whole animal |  | |  | + (poor quality sequence) | | - |
| Oligochaeta |  |  |  |  |  | |  |  | |  |
| (none) | Naididae | Naididae sp. | whole animal | NA | + (MG976145) | | NA |  | |  |
|  | Enchytraeidae | Enchytraeidae sp. | whole animal | NA | + (MG976145) | | NA |  | |  |
|  |  | unknown | whole animal | NA | + (poor quality sequence) | | NA |  | |  |
|  |  | unknown | whole animal | NA | + (poor quality sequence) | | NA |  | |  |
|  |  | unknown | whole animal | NA | + (poor quality sequence) | | NA |  | |  |
| Hirudinea |  |  |  |  |  | |  |  | |  |
| (none) | Glossiphoniidae | *Alboglossiphonia* sp. | whole animal | NA | + (MG976140) | | NA |  | |  |
| Bivalvia |  |  |  |  |  | |  |  | |  |
| (shell, soft bodied) | Sphaeriidae | *Pisidium* sp. | whole animal | whole animal | + | | NA |  | |  |
|  | Sphaeriidae | *Pisidium* sp. | whole animal | whole animal | + | | - |  | |  |
|  | Sphaeriidae | *Pisidium* sp. | whole animal | whole animal | - | | - |  | |  |
| Gastropoda |  |  |  |  |  | |  |  | |  |
| (shell, soft bodied) | Hydrobiidae | *Victodrobia* sp. | whole animal | whole animal |  | |  | + (poor quality sequence) | | - |
|  | Lymnaeidae | *Pseudosuccinea columella* | whole animal | whole animal |  | |  | + (MG976150) | | + (MG976150) |
|  | Lymnaeidae | *Pseudosuccinea columella* | whole animal | whole animal |  | |  | + (MG976215) | | + (MG976214) |
| Diptera |  |  |  |  |  | |  |  | |  |
| (variable usually only head capsule if present) | Ceratopogonidae | Ceratopogonidae sp. | whole animal | tissue |  | |  | + (MG976129) | | + (poor quality sequence) |
|  | Ceratopogonidae | Ceratopogonidae sp. | whole animal | tissue |  | |  | +(MG976130) | | + (MG976131) |
|  | Ceratopogonidae | Ceratopogonidae sp. | whole animal | tissue |  | |  | - | | - |
|  | Ceratopogonidae | Ceratopogonidae sp. | whole animal | tissue |  | |  | + (MG976132) | | + (poor quality sequence) |
|  | Chironomidae | *Riethia* sp. | whole animal | tissue |  | |  | + (MG976133) | | + (MG976134) |
|  | Chironomidae | Orthocladiinae sp. | whole animal | tissue |  | |  | + | | + |
|  | Chironomidae | *Thienemanniella* sp. | whole animal | tissue |  | |  | + (MG976123) | | - |
|  | Muscidae | Muscidae sp. | whole animal | tissue |  | |  | + | | - |
|  | Dixidae | Dixidae sp. | whole animal | tissue |  | |  | + | | - |
|  | Simuliidae | *Austrosimulium* sp. | whole animal | tissue |  | |  | + (MG976158) | | - |
|  | Tipulidae | Tipulidae sp. | whole animal | tissue |  | |  | + (MG976169) | | - |
|  | Tipulidae | *Limnophila* sp. | whole animal | tissue |  | |  | + | | - |
| Trichoptera |  |  |  |  |  | |  |  | |  |
| ((Moderate confined to head, thorax and legs) | Calocidae | *Tamasia acuta* | whole animal | Leg |  | |  | + (MG976085) | | + (MG976086) |
|  | Hydrobiosidae | *Hydrobiosella perangusta* | whole animal | Leg |  | |  | + (MG976211) | | + (MG976210) |
|  | Hydropsychidae | *Asmicridea* sp. | whole animal | Leg |  | |  | + (MG976172) | | + (MG976173) |
|  | Hydrobiosidae | *Hydrobiosella perangusta* | whole animal | Leg |  | |  | + (MG976159) | | + |
|  | Leptoceridae | *Notalina ordina* | whole animal | Leg |  | |  | + (MG976161) | | + (MG976162) |
|  | Leptoceridae | *Triplectides proximus* | whole animal | Leg |  | |  | + (MG976213) | | + (MG976212) |
|  | Leptoceridae | *Triplectides proximus* | whole animal | Leg |  | |  | + (MG976160) | | + |
|  | Philorheithridae | *Kosrheithrus* sp. | whole animal | Leg |  | |  | + (MG976164) | | + (MG976163) |
| Ephemeroptera |  |  |  |  |  | |  |  | |  |
| (moderate whole body) | Baetidae | unknown | whole animal | Leg |  | |  | - | | + (poor quality sequence) |
|  | Baetidae | unknown | whole animal | Leg |  | |  | + (MG976110) | | + (MG976111) |
|  | Leptophlebiidae | *Austrophlebioides marchanti* | whole animal | Leg |  | |  | + (MG976146) | | + (MG976147) |
|  | Leptophlebiidae | *Austrophlebioides marchanti* | whole animal | Leg |  | |  | + (MG976148) | | - |
|  | Leptophlebiidae | *Atalophlebia* sp. | whole animal | Leg |  | |  | + | | + |
|  | Leptophlebiidae | *Nousia* sp. | whole animal | Leg |  | |  | + (MG976125) | | + (MG976126) |
| Plecoptera |  |  |  |  |  | |  |  | |  |
| moderate whole body) | Gripopterygidae | *Acruroperla* sp. | whole animal | Leg |  | |  | + (MG976156) | | + (MG976157) |
|  | Gripopterygidae | *Riekoperla rugosa* | whole animal | Leg |  | |  | + | | + |
|  | Eustheniidae | *Cosmioperla* sp. | whole animal | Leg |  | |  | + (MG976148) | | + (MG976149) |
|  | Eustheniidae | *Cosmioperla* sp. | whole animal | Leg |  | |  | + | | + |
| Hemiptera |  |  |  |  |  | |  |  | |  |
| (moderate/ heavy whole body) | Corixidae | *Sigara* sp. | whole animal | Leg |  | |  | + (MG976119) | | + (MG976120) |
|  | Corixidae | Corixidae sp. | whole animal | Leg |  | |  | + | | + |
|  | Gerridae | Gerridae sp. | whole animal | Leg |  | |  | + (MG976138) | | + (MG976139) |
|  | Notonectidae | Notonectidae sp. | whole animal | Leg |  | |  | + | | + |
| Coleoptera |  |  |  |  |  | |  |  | |  |
| (heavy in adults/ moderate in larvae) | Elmidae | *Notriolus* sp. Larvae | whole animal | Leg |  | |  | + | | + |
|  | Gyrinidae | *Aulonogyrus strigosus* Adult | whole animal | Leg |  | |  | + (MG976141) | | + (MG976142) |
|  | Elmidae | Elmidae sp. Larvae | whole animal | Leg |  | |  | + (MG976115) | | + (MG976116) |
|  | Ptilodactylidae | *Byrrocryptus* sp. Larvae | whole animal | Leg |  | |  | + (MG976152) | | + (MG976153) |
|  | Psephenidae | *Sclerocyphon* sp. Larvae | whole animal | Leg |  | |  | + (MG976204) | | + (MG976205) |
|  | Psephenidae | *Sclerocyphon* sp. Larvae | whole animal | Leg |  | |  | + (MG976206) | | + (MG976207) |
|  | Scirtidae | Scirtidae sp. Larvae | whole animal | Leg |  | |  | + (MG976154) | | + (MG976155) |
|  | Scirtidae | Scirtidae sp. Larvae | whole animal | Leg |  | |  | + | | + |
| Amphipoda |  |  |  |  |  | |  |  | |  |
| (moderate whole body) | Chiltoniidae | *Austrochiltonia subtenuis* | whole animal | Leg |  | |  | + | | + |
|  | Chiltoniidae | *Austrochiltonia subtenuis* | whole animal | Leg |  | |  | + | | + |
|  | Paracalliopiidae | *Paracalliope* sp. | whole animal | Leg |  | |  | + (MG976106) | | + (MG976107) |
|  | Paracalliopiidae | *Paracalliope* sp. | whole animal | Leg |  | |  | + | | + |
| Odonata |  |  |  |  |  | |  |  | |  |
| (Moderate whole body) | Coenagrionidae | *Ischnura heterosticta* small nymph | whole animal | Leg |  | |  | + (MG976117) | | + (MG976118) |
| Megaloptera |  |  | whole animal |  |  | |  |  | |  |
| (moderate confined to head, thorax and legs) | Corydalidae | *Archichauliodes* sp. larvae | whole animal | Leg |  | |  | + (MG976208) | | + (MG976209) |
| Decapoda |  |  |  |  |  | |  |  | |  |
| (moderate whole body) | Atyidae | *Paratya australiensis* juvenile | whole animal | Leg |  | |  | + (MG976127) | | + (MG976128) |
|  | Parastacidae | *Cherax destructor* juvenile | whole animal | Leg |  | |  | + (MG976122) | | + (MG976123) |
