# Supplementary material for: Can non-destructive DNA extraction of bulk invertebrate samples be used for metabarcoding?
Source: PeerJ. 2018 Jun 13;6:e4980. doi: 10.7717/peerj.4980 (PMC6004113; doi:10.7717/peerj.4980)
Supplement: Table S3 — The sample which the individual was taken and the family and species are also given. [file peerj-06-4980-s003.docx]

| Sample | GenBank accession no. | Family | Species |
| --- | --- | --- | --- |
| Sample A | MG976085 | Calocidae | *Tamasia acuta* |
| Sample A | MG976090 | Dytiscidae | *Necterosoma penicillatum* |
| Sample B | MG976086 | Calocidae | *Tamasia acuta* |
| Sample B | MG976087 | Chironomidae | *Cladotanytarsus australomancus* |
| Sample B | MG976088 | Corixidae | *Agrapocorixia eurynome* |
| Sample B | MG976089 | Dytiscidae | *Necterosoma penicillatum* |
| Sample B | MG976091 | Elmidae | Elmidae sp. |
| Sample B | MG976092 | Leptophlebiidae | *Nousia* sp. |
| Sample B | MG976217 | Chiltoniidae | *Austrochiltonia subtenuis* |
| Sample B | MG976093 | Simuliidae | *Simulium ornatipes* |
| Sample C | MG976094 | Paracalliopidae | *Paracalliope* sp. |
| Sample C | MG976095 | Corixidae | *Micronecta* sp. |
| Sample C | MG976096 | Gripopterygidae | *Dinotoperla thwaites* |
| Sample C | MG976097 | Hydrobiosidae | *Ethochorema turbidum* |
| Sample C | MG976098 | Chironomidae | *Cladotanytarsus australomancus* |
| Sample C | MG976099 | Physidae | *Physa acuta* |
| MCL 2016 | MG976175 | Chironomidae | *Polypedilum nubifer* |
| MCL 2016 | MG976176 | Ecnomidae | *Ecnomus continentalis* |
| MCL 2016 | MG976177 | Hydroptilidae | *Hellyethira simplex* |
| MOH 2014 | MG976180 | Corixidae | *Micronecta* sp. |
| MOH 2014 | MG976181 | Chiltoniidae | *Austrochiltonia subtenuis* |
| MOH 2016 | MG976182 | Baetidae | *Offadens* sp. |
| MOH 2016 | MG976183 | Caenidae | *Tasmanocoenis* sp. |
| MOH 2016 | MG976185 | Ecnomidae | *Ecnomus pansus* |
| MOH 2016 | MG976186 | Coenagrionidae | *Ischnura heterosticta* |
| MOH 2016 | MG976187 | Physidae | *Physa acuta* |
| MOH 2016 | MG976188 | Veliidae | *Microvelia* sp. |
| MRD 2016 | MG976189 | Planorbidae | Planorbidae sp. |
| MRD 2016 | MG976190 | Scritidae | *Scrites exoletus* |
| MRD 2016 | MG976191 | Paracalliopidae | *Paracalliope* sp. |
| MRD 2016 | MG976192 | Chironomidae | *Cladotanytarsus* sp. |
| MRD 2016 | MG976193 | Chironomidae | *Chironomus cloacalis* |
| MRD 2016 | MG976194 | Chironomidae | *Polypedilum vespertinus* |
| MRD 2016 | MG976195 | Chironomidae | *Cryptochironomus* sp. |
| MRD 2016 | MG976196 | Corixidae | *Sigara* sp. |
| MRD 2016 | MG976197 | Hydrobiidae | *Austropyrgus* sp. |
| MRD 2016 | MG976198 | Glossiphoniidae | *Alboglossiphonia* sp. |
| MRD 2016 | MG976199 | Glossiphoniidae | *Alboglossiphonia* sp. |
| MRD 2016 | MG976200 | Stratiomyidae | Stratiomyidae sp. |
