# Supplementary material for: Can non-destructive DNA extraction of bulk invertebrate samples be used for metabarcoding?
Source: PeerJ. 2018 Jun 13;6:e4980. doi: 10.7717/peerj.4980 (PMC6004113; doi:10.7717/peerj.4980)
Supplement: Table S4 [file peerj-06-4980-s004.docx]

| Site code | Site location | Latitude | Longitude |
| --- | --- | --- | --- |
|  |  |  |  |
| MCL | Merri Creek at Coburg lake, Coburg, Melbourne, Victoria, Australia. | -37.4385 | 144.5806 |
| MOH | Merri Creek at O'Herns Rd, Broadmeadows, Melbourne, Victoria, Australia. | -37.3786 | 144.5747 |
| MRD | Merri Creek at Rushwood Dr, Craigieburn, Melbourne, Victoria, Australia. | -37.6130 | 144.9518 |
