# Supplementary figures and images for: Can non-destructive DNA extraction of bulk invertebrate samples be used for metabarcoding?
Source: PeerJ. 2018 Jun 13;6:e4980. doi: 10.7717/peerj.4980 (PMC6004113; doi:10.7717/peerj.4980)

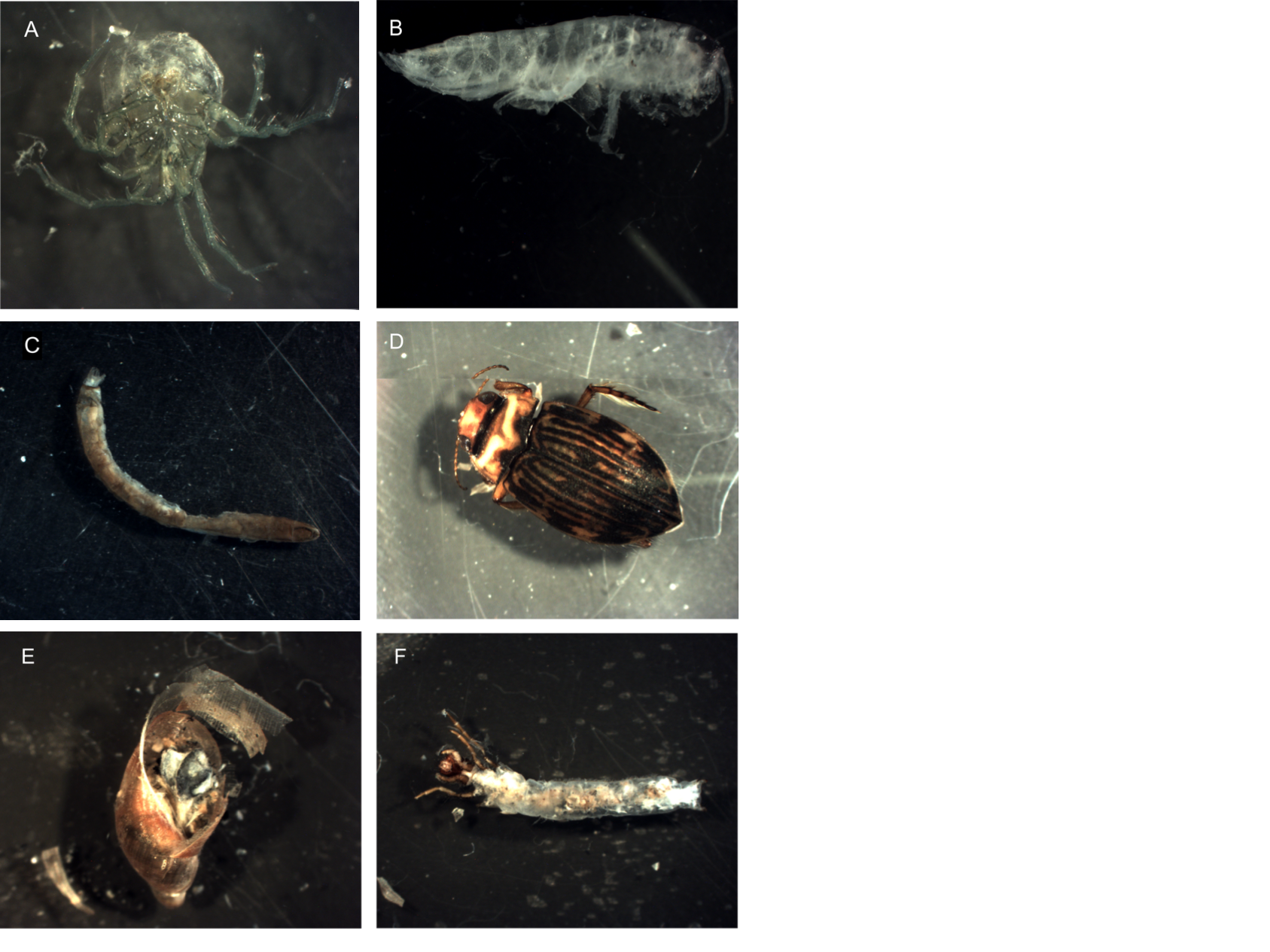

Supplement: Figure S1 — (A) Acarina (Hydrachnidae), (B) Amphipoda (Chiltoniidae), (C) Diptera (Tipulidae), (D) Coleoptera (Dytiscidae), (E) Gastropoda (Lymnaeidae) and (F) Trichoptera (Calocidae). Photos by Melissa Carew. [file peerj-06-4980-s005.png]

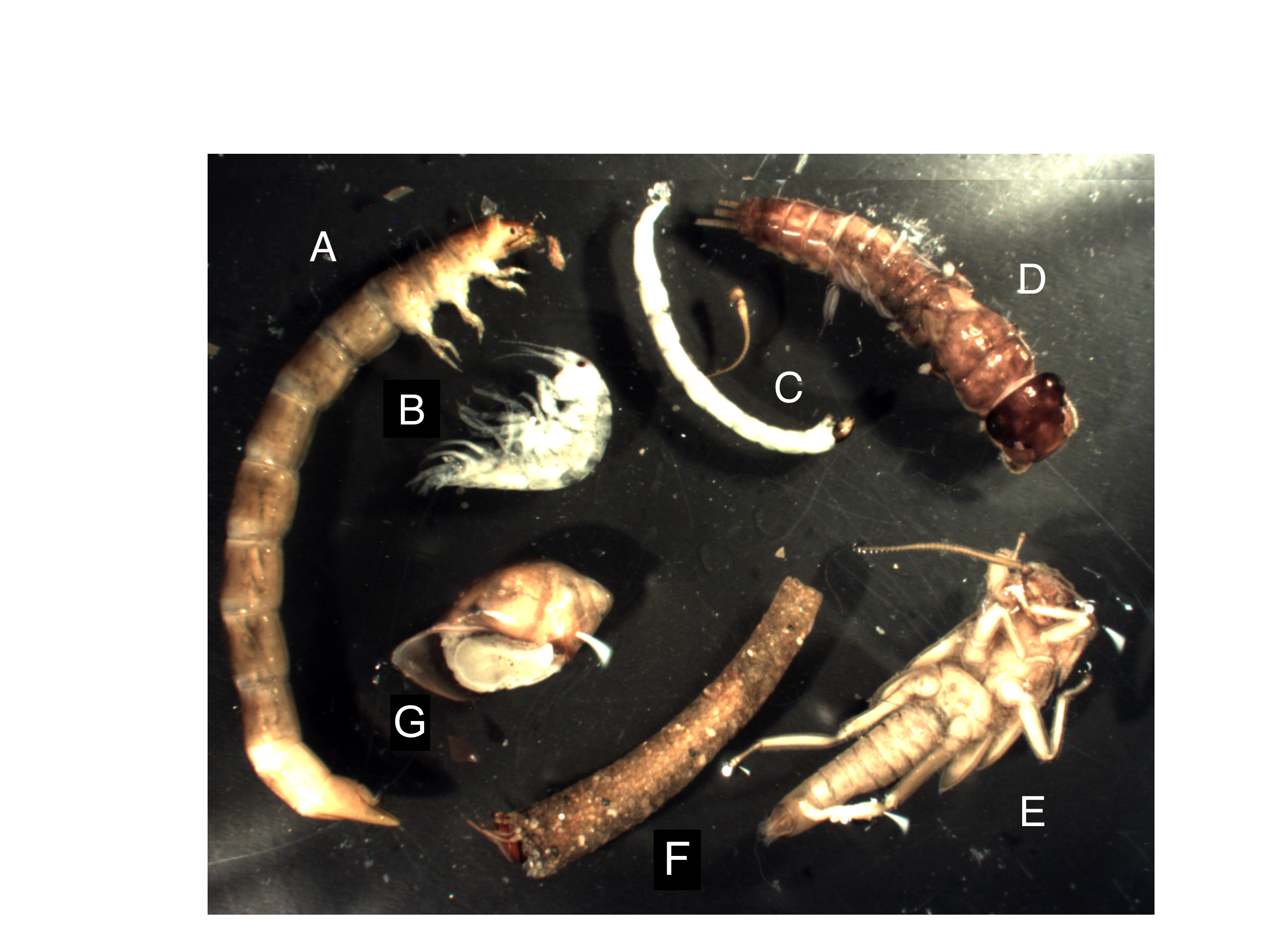

Supplement: Figure S2 — (clockwise) from (A) Coleoptera (Ptilodactylidae), (B) Amphipoda (Chiltoniidae), (C) Diptera (Chironomidae), (D) Ephemeroptera (Leptophlebiidae), (E) Plectoptera (Gripopterygidae), (F) Trichoptera (Calocidae) and (G) Gastropoda (Physidae) after bulk non-destructive DNA extraction for HTS showing that taxa are largely intact for morphological examination and tissue remains for second DNA extraction. In particular, Amphipoda is completely intact after bulk extraction. Photo by Melissa Carew. [file peerj-06-4980-s006.jpg]
